# Proportion and factors influencing client satisfaction with delivery services in health facilities in the Sissala East Municipality, Ghana: A cross‐sectional study

**DOI:** 10.1002/hsr2.1166

**Published:** 2023-03-29

**Authors:** Alijata Braimah, Gifty A. Aninanya, Ebenezer Senu

**Affiliations:** ^1^ Department of Midwifery Midwifery Training College Tumu Upper West Region Ghana; ^2^ Department of Health Services Policy, Planning, Management and Health Economics, School of Public Health University for Development Studies Tamale Northern Region Ghana; ^3^ Department of Molecular Medicine, School of Medicine and Dentistry Kwame Nkrumah University of Science and Technology Kumasi Ghana

**Keywords:** client satisfaction, delivery outcome, delivery services, kind of delivery, maternal health, Tumu municipality

## Abstract

**Background and Aims:**

Client satisfaction is the difference between the healthcare services delivered and the needs of the client. Anecdotal evidence suggests the quality of maternal health and delivery services in Ghana especially in the Upper West Region is appalling. Moreover, there is a paucity of data on clients' satisfaction with maternal and delivery services rendered by healthcare. This study, therefore, assessed clients' satisfaction with delivery services and their associated factors.

**Methods:**

This analytical cross‐sectional study included 431 women who had delivered in the last 7 days from four health facilities within Sissala East Municipality using a multistage and simple random sampling technique. A well‐structured questionnaire was used to collect sociodemographic and client satisfaction data. All statistical analyses were done using Statistical Package for Social Sciences Version 26.0 and GraphPad Prism Version 8.0. A *p* < 0.05 was considered statistically significant.

**Results:**

Clients’ satisfaction with general delivery services was rated as 80.3% and was significantly associated with process‐related factors (*p* < 0.0001) and structural‐related factors (*p* < 0.0001) of the health facilities. This study found that health facilities' delivery services differed significantly and were associated with clients’ satisfaction (*p* < 0.0001). Moreover, age group (*p* = 0.0200), occupation (*p* = 0.0090), kind of delivery (*p* = 0.0050), and delivery outcome (*p* < 0.0001) were significantly associated with client satisfaction with delivery services.

**Conclusion:**

More than two‐thirds of women are satisfied with delivery services within selected health facilities in the Sissala East municipality, although satisfaction within health facilities differs. Furthermore, age group, occupation, kind of delivery, delivery outcome, process, and structural‐related factors significantly contribute to client satisfaction with delivery services. To provide more comprehensive coverage of customers' satisfaction with delivery services in the municipality, strategies such as free maternal health initiatives and health education on the significance of facility delivery should be reinforced.

## 1 INTRODUCTION

Globally, maternal deaths have witnessed a significant reduction compared to maternal deaths in the year 2000. An estimated number of 495,000 maternal deaths were recorded globally, and the 2017 global maternal deaths were estimated to be 279,000 (35%).^1^ Moreover, in 2017, an estimated 515,000 pregnancy‐related deaths were recorded globally, and 30 million women suffered from maternal‐related complications in developing countries yearly.^2^ Over 98% of these maternal deaths were recorded in resource‐poor countries.^3^ Currently, the African Region accounts for 65% (two‐thirds) of maternal deaths in low‐ and middle‐income countries.^1^ In Ghana, maternal mortality is reported to have decreased sharply from 760/100,000 live births in 1990 to 319 per every 100,000 live births in 2015.^4^

Previous studies showed that preterm birth and stillbirths are associated with maternal problems, such as hypertensive disorders of pregnancy, nonobstetric complications, obstetric hemorrhage, and pregnancy‐related infections.^5^, ^6^ Reduction in maternal mortality can be achieved by making health services available, accessible, and of quality to women in cases of complications related to pregnancy and childbirth.^7^, ^8^, ^9^ The most common evidence‐based practice for improving quality care includes active referral and actionable information systems, adequate flow of communication, respect, dignity preservation, supporting women emotionally, engagement of talented and motivated human resources, and making available essential physical infrastructure or resources.^10^

Client satisfaction could be viewed as the difference between the healthcare services delivered and the needs of the client. This measurement gives an opportunity to reduce maternal mortality due to the fact that the mothers will obey the instructions of the healthcare provider once they are satisfied with the delivery of care which meets their needs, and health authorities use the feedback from clients to improve upon service delivery which will ultimately help in reducing mortalities.^11^ Furthermore, client satisfaction is the evaluation of healthcare received. It is usually based on their perception of the health service rendered to them and the physical structures and relationships which exist between health providers, the performance of their roles, while others see clients' satisfaction as a comparison between their expectations and what they experienced.^12^, ^13^

Ghana has made some gains toward addressing maternal and under‐five (5) mortality during the Millenium Development Goals (MDGs) era. Currently, maternal mortality stands at 310 in every 100,000 live births and 52/1000 live births for children under‐five (5) years.^5^ Antenatal coverage nationally in 2018 was 89%, while pregnant women who received skilled birth delivery accounted for 79%.^5^ Attitudes portrayed by healthcare providers, facility characteristics, and sociodemographic characteristics of pregnant women seeking care have been linked to facility delivery services.^14^, ^15^, ^16^

However, evidence suggests that the quality of maternal health services and delivery services especially in the Upper West Region of Ghana is appalling although the region recorded 87% in antenatal care (ANC) and 68.7% in supervised deliveries in 2018.^17^ Recent unpublished data from the Municipal Health Directorate of Sissala East shows that ANC attendance for 2019 and 2018 was 2439 and 2805, respectively, whereas deliveries for the same respective years were also recounted to be 2272 and 2456, respectively.^18^ Stillbirths for 2019 and 2018 were also recorded as 20 and 22, respectively.^18^ In addition, there is a paucity of data on clients’ satisfaction with maternal and child health services rendered by healthcare workers in health facilities in the Upper West Region, including the Sissala East Municipality. Anecdotal evidence suggests that some pregnant women are not happy with some of the maternal and child health services received. The consequences of pregnant women not being attended to or served by skilled healthcare providers could result in complications for the mother and babies. In the unfortunate event, maternal and neonatal deaths may occur and hence the need to explore the factors accounting for clients' satisfaction with delivery services in the municipality.

## 2 MATERIALS AND METHODS

### 2.1 Study design

In achieving the main objective of the study, an analytical cross‐sectional design was adopted using the quantitative approach. The design was used to determine the factors that influence client satisfaction with care in a specific population at a single moment in time.^19^

### 2.2 Study site

The study was conducted in four health facilities such as Tumu Municipal Hospital, Wellembelle Health Center, Sakai Health Center, and Kulfuo Health Center within Sissala East Municipality. The Sissala municipality is located in Ghana's Upper West Region's northeastern enclave. It is located between 1.300 W longitude and 10.000 N and 11.000 N latitude and shares a border with Burkina Faso on the north, Kassena‐Nankana West, and Builsa District on the east, West Mamprusi District on the south, Wa East, and Daffiama‐Bussie‐Issah districts on the south, and Sissala West District on the west. About 4 out of 10 people aged 12 and above are married (52.7%), 1% are divorced, and 0.8% are separated. By the age of 25–29, moreover, half of the females (79.8%) are married, compared to just under half of males (44.4%). Previous statistics showed 78.5% of married adults have never attended school, compared to 30.8% of unmarried people.^20^ Tumu which is the capital and has a municipal hospital which is the only referral facility for the municipality and receives referrals from the Sissala West district.

### 2.3 Study population

This study included all women who had delivered in the last 7 days and between the age brackets of 18–49 years at the time of conducting the study in the four selected health facilities. Exclusion criteria include pregnant women in labor. Postnatal mothers who had exceeded the seventh day following delivery and had reported to the facility were also excluded from the study.

### 2.4 Sample size calculation

The women of fertility age population in the district is estimated as 16,087. The sample size was then obtained by Yamane's formula for a random sample size of a known population.^21^

$n=\frac{N}{1+N{\left(e\right)}^{2}}$, where *n* is the minimum sample size required, *N* is the estimated population (16,087), *e* is the margin of error (5%) at a 95% confidence interval

$$n=\frac{\;16087}{1+16087{\left(0.05\right)}^{2}}=390.3(390).$$

Hence, a minimum of 390 participants were required for the study. Accounting for 10% nonresponse of the respondents and to increase statistical power, 431 women were recruited for the study.

### 2.5 Sampling technique

Multistage sampling and simple random sampling were used to employ in selecting the submunicipals and health centers within the submunicipals, respectively. Thus, at the municipal level, the Tumu Municipal Hospital, which is the main referral facility, was purposively selected for the study. The municipal has seven  submunicipals through which it provides healthcare services to individuals within its catchment area and to ensure that some important characteristics of the population are fairly represented, a simple random sampling through balloting without replacement was used to select three submunicipals from the seven submunicipal. This was done through balloting where the names of all submunicipals were written on paper, folded, and placed in one bowl for the rural submunicipal and the other bowl for the urban submunicipality. With one's eyes closed, two submunicipals from the rural bowl and one from the urban submunicipals were picked. Next was picking two health facilities under the already chosen two rural submunicipal and picking one health facility in the urban submunicipal category from the one that was selected. The facilities for the study include the Tumu Municipal Hospital and other selected health centers. Respondents in these facilities were selected consecutively provided they accepted to participate in the study and sign the consent form.

### 2.6 Instruments and procedures for data collection and measurement of client satisfaction

Instruments for data collection comprised a structured questionnaire developed based on studies done earlier among delivery clients.^14^, ^22^ The primary data included raw data collected using questionnaires which were done by the researcher and four research assistants.

The questionnaire consists of questions related to the rating of hygiene of the facility, the attitude of healthcare providers toward clients, the competence of care providers, general health information on labor and 7 days postnatal, and client–healthcare provider interaction on the sociodemographic characteristics information of respondents was also obtained. All clients who came to the selected facilities were questioned once they accepted to partake in the study and after signing the agreement form. The questionnaires were developed in English before translating into Sissali language and back to English by Mr. Batong Khalid of SILDEP, nongovernmental organization.

Data collection was carried out at the health facility. Four hundred and thirty‐one questionnaires were administered to the respondents in four health facilities where labor and delivery services are conducted. Data collection started on February 24, 2021 after obtaining ethical approval on February 11, 2021 and ended in April 2021. An average of 30 min was spent on each questionnaire. The questionnaires were administered in English, Sissali, or the Dagaare language.

The researcher entered selected health facilities to identify postnatal mothers who delivered day 1 up to the 7th day by questioning them on the date of delivery and postnatal day. Eligible participants were selected from each facility. The researcher explained the purpose of the study in the preferred language of the respondent to gain their consent and cooperation to participate in the study.

### 2.7 Measures for quality control

A pretest was conducted among 20 respondents in the Nabulo subdistrict who were not part of the study. This facility that was chosen shared similar characteristics with the respondents who were used in the study. This was done to confirm the appropriateness of the data collection tools. It was tested for clarity, and construction of the questions, and questions that are not clear will be revised. The principal researcher monitored the research team to ensure that the interviews were well conducted in the selected study areas.

Four research assistants were recruited and trained to assist the researcher in the administration of the questionnaire. Due diligence was done to ensure that the assistants collected complete data on each respondent. All returned questionnaires on each day were checked for completeness and data were entered into Microsoft Excel 2019 for analysis.

### 2.8 Data management and statistical analysis

Collected data were entered, cleaned, and coded using Microsoft Excel 2019. All statistical analyses were done using the Statistical Package for Social Sciences Version 26.0, and GraphPad Prism Version 8.0 (GraphPad Software, www.graphpad.com). All categorical variables were represented as frequency and percentages. Bar charts were used to represent the proportion of the general client satisfaction process and structural‐related client satisfaction at delivery. A *χ* ^2^ test statistic was used to determine the factors associated with general process and structural‐related client satisfaction at delivery among study participants. A *p‐*value of <0.05 was considered statistically significant.

## 3 RESULTS

### 3.1 Sociodemographic characteristics of study participants

Of the 431 women recruited using multistage and simple random sampling techniques and included in the statistical analyses, more than half were from Tumu Municipal Hospital (51.0%), 25.5% were from Kulfuo Health Center, 14.4% were from Wellembelle Health Center, and 9.0% were from Sakai Health Center. Most were within 18–22 years (33.6%), followed by 28–32 years (27.8%), 23–27 years (26.2%), and a few were 38–42 years (2.6%) and 43–47 years (0.3%). Over 90% (90.3%) were married and most had given birth multiple times (60.3%) with 39.7% who had given birth for the first time. Moreover, about one‐quarter had never attended school (26.2%), while more than half had primary or secondary education (52.2%), with some who had tertiary education (15.8%), vocational, or technical skills (5.8%). Most participants were farmers (32.9%) and housewives (28.8%) with an average monthly income of Gh₵100.00 to Gh₵300.00 (83.3%). The majority of women had a normal vaginal delivery (82.4%), with some who had planned cesarean delivery (8.4%), an emergency cesarean delivery (6.5%), and vaginal delivery assisted by equipment (2.8%) (Table 1).

**Table 1:** Sociodemographic characteristics of study participants.

Variable	Frequency (*n* = 431)	Percentage (%)
Number of deliveries		
Tumu Municipal Hospital	220	51.0
Wellembelle Health Center	62	14.4
Sakai Health Center	39	9.0
Kulfuo Health Center	110	25.5
Age groups (years)		
18–22	145	33.6
23–27	113	26.2
28–32	120	27.8
33–37	41	9.5
38–42	11	2.6
43–47	1	0.3
Marital status		
Single	42	9.7
Married	389	90.3
Parity		
Primipara	171	39.7
Multipara	260	60.3
Educational level		
Never attended school	113	26.2
Primary/secondary	225	52.2
Vocational/technical	25	5.8
Tertiary	68	15.8
Occupation		
Trader	47	10.9
Farmer	142	32.9
Housewife	124	28.8
Student	51	11.8
Self‐employed	67	15.5
Average monthly income		
100gh–300Gh₵	359	83.3
400gh–600Gh₵	49	11.4
700gh–900Gh₵	12	2.8
1000gh and above	11	2.6
Kind of delivery		
Normal vaginal delivery	355	82.4
Vaginal delivery assisted by equipment	12	2.8
Planned cesarean delivery	36	8.4
Emergency cesarean delivery	28	6.5
Outcome of delivery		
Normal	397	92.1
With complications	34	7.9

*Note*: Data presented as frequencies and percentages.

### 3.2 Proportion of general client satisfaction with delivery services

**Figure 1:**
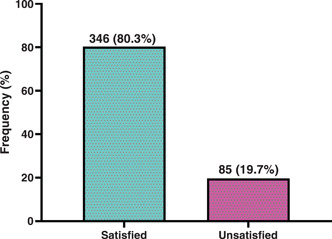
Proportion of general client satisfaction with delivery services in health facilities in the Sissala East Municipality, Ghana.

Respondents were asked to rate their general satisfaction with the delivery services in the various health facilities, the majority of them rated it as satisfactory (80.3%) while 19.7% rated as unsatisfactory (Figure 1).

### 3.3 Proportion client satisfaction stratified by process‐ and structural‐related factors during delivery services

**Figure 2:**
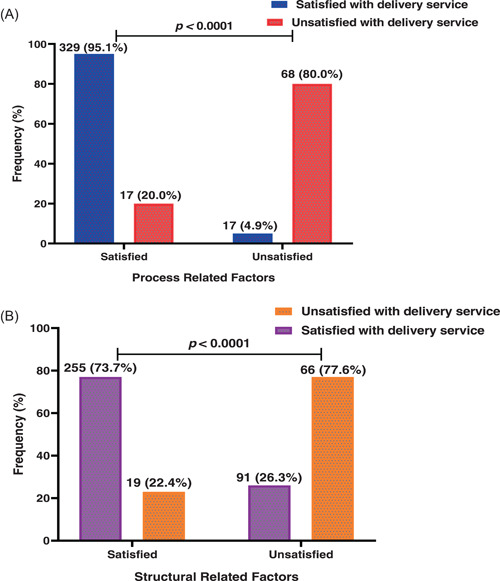
Proportion client satisfaction stratified by process (A) and structural (B) related factors during delivery services among women in health facilities in the Sissala East Municipality, Ghana.

Of 346 women who were satisfied with general delivery services, over 90% were satisfied with process‐related factors (95.1%). Of 85 women unsatisfied with general delivery services, majority (80.0%) were unsatisfied with process‐related factors. A significant association was, therefore, observed between satisfaction with general delivery services and that of process‐related factors (*p* < 0.0001) (Figure 2A).

Moreover, this study found a significant association between women satisfaction with general delivery services and structural‐related factors of health facilities (*p* < 0.0001). This is due to over three‐quarter women (77.6%) among those unsatisfied with general delivery services that were equally unsatisfied with structural‐related factors (*p* < 0.0001) (Figure 2B).

### 3.4 Factors associated with client satisfaction during delivery services

This study found that the health facilities within the Sissala East Municipality delivery services differ and were significantly associated with participant satisfaction (*p* < 0.0001). Moreover, the age group of study participants (*p* = 0.0200) and occupation (*p* = 0.0090) were significantly associated with satisfaction with delivery services. Similarly, the kind of delivery (*p* = 0.0050) and delivery outcome (*p* < 0.0001) were significantly associated with general satisfaction with delivery services.

However, no significant association was found between the marital status of women (*p* = 0.1800), parity (*p* = 0.0960), educational level (*p* = 0.1350), women's average monthly income (*p* = 0.9160), and satisfaction with delivery services (Table 2).

**Table 2:** Factors associated with client satisfaction during delivery services.

Satisfaction with delivery services			
Variable	Satisfied (*n* = 346)	Unsatisfied (85)	*p* Value
Facility name			**<0.0001**
Tumu Municipal Hospital	154 (44.5)	66 (77.6)	
Wellembelle Health Center	59 (17.1)	3 (3.5)	
Sakai Health Center	36 (10.4)	3 (3.5)	
Kulfuo Health Center	97 (28.03)	13 (15.4)	
Marital status			0.1800
Single	37 (10.7)	5 (5.9)	
Married	309 (89.3)	80 (94.1)	
Age group (years)			**0.0200**
Less than 20	48 (13.8)	6 (7.1)	
20–29	211 (61.0)	44 (51.8)	
30–39	83 (24.0)	33 (38.8)	
40–49	4 (1.2)	2 (2.4)	
Parity			0.0960
Primipara	144 (41.6)	27 (31.8)	
Multipara	202 (58.4)	58 (68.2)	
Educational level			0.1350
Never attended school	98 (28.3)	18 (21.2)	
Primary/secondary	177 (51.2)	48 (56.5)	
Vocational/technical	18 (5.2)	4 (4.7)	
Tertiary	53 (15.3)	15 (17.6)	
Occupation			**0.0090**
Trader	29 (8.4)	18 (21.2)	
Farmer	122 (35.3)	20 (23.5)	
Housewife	101 (29.2)	23 (27.1)	
Student	42 (12.1)	9 (10.6)	
Self‐employed	52 (15.0)	15 (17.6)	
Average monthly income			0.9160
100gh–300gh	290 (83.8)	69 (81.2)	
400gh–600gh	38 (11.0)	11 (12.9)	
700gh–900gh	9 (2.6)	3 (3.5)	
1000gh and above	9 (2.6)	2 (2.4)	
Kind of delivery			**0.0050**
Normal vaginal delivery	295 (85.3)	60 (70.6)	
Vaginal delivery assisted by equipment	6 (1.7)	6 (7.1)	
Planned cesarean delivery	26 (7.5)	10 (11.8)	
Emergency cesarean delivery	19 (5.5)	9 (10.6)	
Delivery outcome			
Normal	331 (95.7)	66 (77.6)	**<0.0001**
With complications	15 (4.3)	19 (22.4)	

*Note*: Data presented as frequencies and percentages; *p* < 0.05 and bolded means statistically significant

### 3.5 Association between processes‐related factors and level of satisfaction with delivery services

Of the process‐related factors, the reception given at the health facility (*p* < 0.0001), the privacy provided during stay in the health facility and delivery (*p* < 0.0001), the level of respect received from the health workers (*p* < 0.0001), and the support given by the health workers (*p* < 0.0001) were significantly associated with the level of general client satisfaction with delivery services at the health facilities. Moreover, the information given by the health workers during labor delivery and after delivery (*p* < 0.0001), the waiting time between arriving at the health facility and seeing a health worker (*p* = 0.0004), and the satisfaction with clinical examination performed by health workers during labor and delivery (*p* < 0.0001) were significantly associated with the level of general client satisfaction with delivery services at the health facilities. Similarly, this study observed the level of assistance, attention and care given during and after delivery (*p* < 0.0001) significantly influenced satisfaction with delivery services at health centers. Thus, each process‐related factor contributes to a level of client satisfaction and must be monitored together for total client satisfaction with delivery services at various health facilities (Table 3).

**Table 3:** Association between processes‐related factors and level of satisfaction with delivery services.

Level of satisfaction with delivery satisfaction					
Variable	Very satisfied (*n* = 186)	Satisfied (*n* = 231)	Neutral (*n* = 13)	Unsatisfied (*n* = 1)	*p* Value
Are you satisfied with the reception given to you at the health facility					**<0.0001**
Very satisfied	90 (48.4)	46 (19.9)	2 (15.4)	0 (0.0)	
Satisfied	90 (48.4)	173 (74.9)	9 (69.2)	0 (0.0)	
Neutral	5 (2.7)	5 (2.2)	2 (15.4)	0 (0.0)	
Unsatisfied	1 (0.5)	5 (2.2)	0 (0.0)	1 (100.0)	
Very unsatisfied	0 (0.0)	2 (0.9)	0 (0.0)	0 (0.0)	
Is your satisfaction ok with the privacy provided during your stay in the health facility and delivery?					**<0.0001**
Very satisfied	89 (47.8)	41 (17.7)	2 (15.4)	0 (0.0)	
Satisfied	89 (47.8)	173 (74.9)	9 (69.2)	1 (100.0)	
Neutral/somewhat satisfied	5 (2.7)	14 (6.1)	2 (15.4)	0 (0.0)	
Unsatisfied	3 (1.7)	3 (1.3)	0 (0.0)	0 (0.0)	
Very unsatisfied	0 (0.0)	0 (0.0)			
Are you satisfied with the level of respect you received from the health workers?					**<0.0001**
Very satisfied	75 (40.3)	42 (18.2)	4 (30.8)	0 (0.0)	
Satisfied	107 (57.5)	169 (73.2)	3 (23.1)	0 (0.0)	
Neutral	4 (4.3)	18 (7.8)	6 (46.2)	0 (0.0)	
Unsatisfied	0 (0.0)	1 (0.4)	0 (0.0)	1 (100.0)	
Very unsatisfied	0 (0.0)	1 (0.4)	0 (0.0)	0 (0.0)	
Are you satisfied with the support given by the health workers?					**<0.0001**
Very satisfied	82 (44.1)	41 (17.7)	1 (7.7)	0 (0.0)	
Satisfied	99 (53.2)	176 (76.2)	7 (53.8)	0 (0.0)	
Neutral	5 (2.7)	13 (5.6)	3 (23.1)	0 (0.0)	
Unsatisfied	0 (0.0)	1 (0.4)	1 (7.7)	1 (100.0)	
Very unsatisfied	0 (0.0)	0 (0.0)	1 (7.7)	0 (0.0)	
Did not receive any drugs	0 (0.0)	0 (0.0)	0 (0.0)	0 (0.0)	
With the information given you by the health workers during labor, delivery, and after delivery					**<0.0001**
Very satisfied	65 (34.9)	51 (22.1)	0 (0.0)	0 (0.0)	
Satisfied	109 (58.6)	169 (73.2)	10 (76.9)	0 (0.0)	
Neutral	8 (4.3)	8 (3.5)	1 (7.7)	0 (0.0)	
Unsatisfied	3 (1.6)	2 (0.9)	1 (7.7)	1 (100.0)	
Very unsatisfied	1 (0.5)	1 (0.4)	1 (7.7)	0 (0.0)	
What was the waiting time between arriving at the health facility and seeing a health worker?					**0.0004**
Far too long	11 (5.9)	22 (9.5)	1 (7.7)	0 (0.0)	
Long	28 (15.1)	40 (17.3)	3 (23.1)	1 (100.0)	
Neutral	41 (22.0)	89 (38.5)	6 (46.2)	0 (0.0)	
I was helped immediately; I did not have to wait	106 (57.0)	80 (34.6)	3 (23.1)	0 (0.0)	
Related to the clinical examination performed by health workers during labor and delivery, were you satisfied with it?					
Very satisfied	77 (41.4)	42 (18.2)	3 (23.1)	1 (100.0)	**<0.0001**
Satisfied	99 (53.2)	174 (75.3)	2 (15.4)	0 (0.0)	
Neutral	6 (3.2)	11 (4.8)	8 (61.5)	0 (0.0)	
Unsatisfied	0 (0.0)	3 (1.3)	0 (0.0)	0 (0.0)	
Very unsatisfied	4 (2.2)	1 (0.4)	0 (0.0)	0 (0.0)	
Satisfaction with the level of assistance by health workers during delivery					**<0.0001**
Very satisfied	73 (39.2)	29 (12.6)	2 (15.4)	0 (0.0)	
Satisfied	107 (57.5)	181 (78.4)	6 (46.2)	0 (0.0)	
Neutral	5 (2.7)	18 (7.8)	5 (38.5)	0 (0.0)	
Unsatisfied	0 (0.0)	1 (0.4)	0 (0.0)	1 (100.0)	
Very unsatisfied	1 (0.5)	2 (0.9)	0 (0.0)	0 (0.0)	
With the attention and care given to your newborn baby after delivery, were you satisfied?					**<0.0001**
Very satisfied	89 (47.8)	33 (14.3)	2 (15.4)	1 (100.0)	
Satisfied	89 (47.8)	177 (76.6)	9 (69.2)	0 (0.0)	
Neutral	6 (3.2)	16 (6.9)	2 (15.4)	0 (0.0)	
Unsatisfied	0 (0.0)	2 (0.9)	0 (0.0)	0 (0.0)	
Very unsatisfied	2 (1.1)	3 (1.3)	0 (0.0)	0 (0.0)	
With the level of attention and care given to you after delivery, are you satisfied with it?					**<0.0001**
Very satisfied	78 (41.9)	38 (16.5)	2 (15.4)	0 (0.0)	
Satisfied	100 (53.8)	170 (73.6)	6 (46.2)	1 (100.0)	
Neutral	7 (3.8)	19 (8.2)	4 (30.8)	0 (0.0)	
Unsatisfied	1 (0.5)	2 (0.9)	0 (0.0)	0 (0.0)	
Very unsatisfied	0 (0.0)	2 (0.9)	1 (7.7)	0 (0.0)	

*Note*: Data are presented as frequencies and percentages; *p* < 0.05 and bolded means statistically significant.

### 3.6 Association between structural‐related factors and level of satisfaction with delivery services

Of the structural‐related factors, there was a significant association observed between the medical examination satisfaction by a health worker (*p* < 0.0001), level of assistance given by the caregiver(s) during the delivery (*p* < 0.0001), satisfaction with drugs prescribed by the health worker(s) (*p* < 0.0001), and level of general client satisfaction with delivery services at the health facilities. Furthermore, drugs provided at the health facility (*p* < 0.0001), satisfaction with medical equipment available at the facility (*p* < 0.0001), and hygiene at the facility (*p* < 0.0001) were significantly associated with the level of general client satisfaction with delivery services. This study's findings show that each structural‐related factor singly affects the level of client satisfaction with delivery services and must be wholesomely enhanced for adequate client satisfaction at various health facilities (Table 4).

**Table 4:** Association between structural‐related factors and level of satisfaction with delivery services.

Level of satisfaction with delivery satisfaction					
Variable	Very satisfied (*n* = 186)	Satisfied (*n* = 231)	Neutral (*n* = 13)	Unsatisfied (*n* = 1)	*p* Value
Medical examination satisfaction by a health worker					**<0.0001**
Very satisfied	77 (41.4)	42 (18.2)	3 (23.1)	1 (100.0)	
Satisfied	99 (53.2)	174 (75.3)	2 (15.4)	0 (0.0)	
Neutral	6 (3.2)	11 (4.8)	8 (61.5)	0 (0.0)	
Unsatisfied	0 (0.0)	3 (1.3)	0 (0.0)	0 (0.0)	
Very unsatisfied	4 (2.2)	1 (0.4)	0 (0.0)	0 (0.0)	
Level of assistance given by caregiver (s) during delivery					**<0.0001**
Very satisfied	73 (39.2)	29 (12.6)	2 (15.4)	0 (0.0)	
Satisfied	107 (57.5)	181 (78.4)	6 (46.2)	0 (0.0)	
Neutral	5 (2.7)	18 (7.8)	15 (115.4)	0 (0.0)	
Unsatisfied	0 (0.0)	1 (0.4)	0 (0.0)	1 (100.0)	
Very unsatisfied	1 (0.5)	2 (0.9)	0 (0.0)	0 (0.0)	
Drugs prescribed by the health worker(s)					**<0.0001**
Very satisfied	49 (26.3)	34 (14.7)	2 (15.4)	1 (100.0)	
Satisfied	100 (53.8)	142 (61.5)	5 (38.5)	0 (0.0)	
Neutral	14 (7.5)	26 (11.3)	2 (15.4)	0 (0.0)	
Unsatisfied	3 (1.6)	5 (2.2)	3 (23.1)	0 (0.0)	
Very unsatisfied	1 (0.5)	3 (1.3)	0 (0.0)	0 (0.0)	
Drugs provided at the health facility?					**<0.0001**
Very satisfied	60 (32.3)	25 (10.8)	2 (15.4)	0 (0.0)	
Satisfied	91 (48.9)	152 (65.8)	5 (38.5)	0 (0.0)	
Neutral	12 (6.5)	21 (9.1)	4 (30.8)	0 (0.0)	
Unsatisfied	2 (1.1)	7 (3.0)	2 (15.4)	1 (100.0)	
Very unsatisfied	0 (0.0)	1 (0.4)	0 (0.0)	0 (0.0)	
Did not receive any drugs	24 (12.9)	25 (10.8)	0 (0.0)	0 (0.0)	
Satisfaction with medical equipment available at the facility					**<0.0001**
Very satisfied	64 (34.4)	32 (13.9)	2 (15.4)	0 (0.0)	
Satisfied	107 (57.5)	164 (71.0)	4 (30.8)	0 (0.0)	
Neutral	11 (5.9)	25 (10.8)	2 (15.4)	0 (0.0)	
Unsatisfied	2 (1.1)	8 (3.5)	4 (30.8)	1 (100.0)	
Very unsatisfied	2 (1.1)	2 (0.9)	1 (7.7)	0 (0.0)	
Hygiene at the facility					**<0.0001**
Very satisfied	82 (44.1)	34 (14.7)	5 (38.5)	0 (0.0)	
Satisfied	90 (48.4)	164 (71.0)	2 (15.4)	0 (0.0)	
Neutral	8 (4.3)	17 (7.4)	4 (30.8)	0 (0.0)	
Unsatisfied	2 (1.1)	11 (4.8)	1 (7.7)	1 (100.0)	
Very unsatisfied	4 (2.2)	5 (2.2)	1 (7.7)	0 (0.0)	

*Note*: Data are presented as frequencies and percentages; *p* < 0.05 and bolded means statistically significant.

## 4 DISCUSSION

This study assessed clients' satisfaction with delivery services and their associated factors. Clients' satisfaction with general delivery services was rated as 80.3% and was significantly associated with process‐related factors and structural‐related factors of the health facilities. This study found that health facility delivery services differ and were significantly associated with participant satisfaction. Moreover, age group, occupation, kind of delivery, and delivery outcome were significantly associated with client satisfaction with delivery services. Of the process‐related factors, the reception given at the health facility, the privacy provided during stay in the health facility and delivery, the level of respect received, the support given, the information given by the health workers during labor, delivery, and after delivery, and waiting time were significantly associated with the level of general client satisfaction with delivery services. Of the structural‐related factors, a significant association was observed between medical examination, level of assistance, satisfaction with drugs prescribed, medical equipment available at the facility, hygiene at the facility, and level of general client satisfaction with delivery services.

Clients' satisfaction with general delivery services rated as 80.3% is in line with what was observed in a comprehensive evaluation of women's satisfaction with maternal services in underdeveloped countries.^23^ The present study could have positive health outcomes for clients as satisfied clients are likely to patronize delivery and related services in the future. The satisfaction with general delivery services was significantly associated with process‐related factors and structural‐related factors of the health facilities.

The age group of study participants and occupation being significantly associated with client satisfaction with delivery services are consistent with the Hoseini et al. study which found age, and occupation as variables that are significant to the maternal level of satisfaction with delivery services.^24^

Moreover, the findings of health facilities within the Sissala East Municipality, the kind of delivery, and the delivery outcome being significantly associated with client satisfaction with delivery services are consistent with the studies of Melese et al. and Karoni et al. who found the type of health facility, the mode of delivery being normal vaginal delivery, vaginal delivery assisted, planned cesarean delivery, or emergency cesarean delivery, and delivery outcomes to be factors significantly influencing client satisfaction with delivery services.^25^, ^26^ On the mode of delivery, Karoni et al. reported that mothers who had normal vaginal delivery had a satisfaction level of 65.6% while mothers with cesarean section delivery were 57.2% satisfied in Bahir Dar city in North West Ethiopia. However, an earlier study by Tesfaye et al. in South West Ethiopia reported that women who had undergone a cesarean section were four times more likely to be satisfied than mothers with normal vaginal delivery.^11^ It could, therefore, be argued that other factors besides the mode of delivery could account for client satisfaction with delivery services.

Furthermore, the study suggests that the satisfaction of clients with delivery services at health facilities is linked to the nature of reception and treatment pregnant women receive in these facilities.^27^ This finding is consistent with the present study in that the reception and support given at the health facility influence clients' satisfaction.

Privacy and confidentiality of clients in a health facility are deemed a relevant indicator of good service delivery and a key determinant of client satisfaction with the service provider as indicated by some studies.^23^, ^26^, ^28^ The current study showed that respondents were satisfied with service delivery due to the privacy and confidentiality clients received in the study area. Gitobu et al. in a study conducted in Kenya, however, found more than half of the respondents felt dissatisfied due to lack of privacy.^29^ This finding confirms the privacy provided during stay in the health facility and delivery are important factors of client satisfaction.

Studies by Srivastava et al. and Odonkor et al. found respect, as well as courtesy, according to clients at health facilities during delivery services by health workers as a hallmark of client satisfaction.^23^, ^30^ This was further strengthened in the current study as over 90% of participants pointed out the respect received from health workers as a major contributor to satisfaction.

The findings of information given by the health workers during labor, delivery, and after delivery, satisfaction with drugs prescribed by the health workers, were significantly associated with the level of general client satisfaction with delivery and are in agreement with the studies of Ampofo et al., Peprah et al., and Panth et al. which showed similar findings contributing to client satisfaction.^31^, ^32^, ^33^ In addition, our study also demonstrated waiting time, medical examination, medical equipment available at the facility, and hygiene at the facility are associated with the level of general client satisfaction with delivery services.

Nonetheless, this quantitative study is limited only to selected health facilities in the Tumu municipality and for that matter, future researchers should include all health facilities (institutions) in the Upper West Region through a mixed‐methods approach. Moreover, delivery clients were the only participants that took part in the study and so future studies should interview both health staff and clients so that the responses provided could be validated. The study was also conducted among delivery clients in the wards of the health facilities selected and so it is possible that the majority of the clients reported clients' satisfaction with the services rendered because they wanted to please the health staff who were around the ward during the survey.

## 5 CONCLUSION

More than two‐thirds of women were satisfied with delivery services within selected health facilities in the Tumu municipality in the Upper West Region, although client satisfaction within health facilities differs. Moreover, age group, occupation, kind of delivery, delivery outcome, process, and structural‐related factors significantly contributed to client satisfaction with delivery services. For more thorough coverage of clients' satisfaction with delivery services in the municipality, interventions such as free maternal health initiatives and health education on the importance of facility delivery should be enhanced.

## AUTHOR CONTRIBUTIONS

**Alijata Braimah**: Conceptualization; data curation; investigation; methodology; resources; writing—original draft; writing—review and editing. **Gifty A. Aninanya**: Conceptualization; resources; supervision; writing—original draft; writing—review and editing. **Ebenezer Senu**: Conceptualization; data curation; formal analysis; methodology; software; writing—original draft; writing—review and editing.

## CONFLICT OF INTEREST STATEMENT

The authors declare no conflict of interest.

## ETHICS STATEMENT

Ethics for the study was approved by the Kwame Nkrumah University of Science and Technology ethics board. An introductory letter was also obtained from the University for Development Studies, which was used in obtaining permission from the Health Directorate of the Upper West Region. Lastly, a component of an informed consent form was explained to the respondents and they agreed to participate in the study, and the form was signed by both the researcher and respondents to ensure confidentiality. Informed consent was obtained from the respondent after the consent form had been explained to the respondents on their rights in taking part in the study. Other aspects such as the privacy and confidentiality of respondents will also be highlighted to them. Respondents were also informed that participation in the study was voluntary and they agreed to participate. Data was stored in the cupboard/computer under keys and lock and only opened when necessary.

## TRANSPARENCY STATEMENT

The lead author Ebenezer Senu affirms that this manuscript is an honest, accurate, and transparent account of the study being reported; that no important aspects of the study have been omitted; and that any discrepancies from the study as planned (and, if relevant, registered) have been explained.

## Data Availability

All data generated or analyzed during this study are included in this article and can be requested from the corresponding author.
